# A nationwide longitudinal investigation on the role of prenatal exposure to infectious diseases on the onset of chronic conditions in children and adolescents in Brazil

**DOI:** 10.12688/wellcomeopenres.22430.1

**Published:** 2024-06-19

**Authors:** Enny S. Paixao, Thiago Cerqueira-Silva, Pilar T.V. Florentino, Orlagh Carroll, Nuria Sanchez Clemente, Deborah A. Lawlor, Rita de Cássia Ribeiro Silva, Laura Cunha Rodrigues, Liam Smeeth, Mauricio L. Barreto

**Affiliations:** 1 Faculty of Epidemiology and Population Health, London School of hygiene and Tropical Medicine, London, UK; 2 Centre for Data and Knowledge Integration for Health, Salvador, Brazil; 3 Centre for Neonatal and Paediatric Infection, St George's University, Saint George's, Saint George, UK; 4 MRC Integrative Epidemiology Unit at the University of Bristol, United Kingdom; Population Health Science, Bristol Medical School, University of Bristol, Bristol, England, UK

**Keywords:** maternal infection, syphilis, zika, dengue and chikungunya, in uterus exposure to infection, non-communicable conditions, children, adolescents, life course

## Abstract

**Background:**

In utero exposure to infections might set the stage for a chain of events leading to a wide spectrum of long-term health outcomes observed in children and adolescents. This proposal aims to investigate whether syphilis, zika, dengue and chikungunya during pregnancy can increase the risk of the offspring developing a non-infectious chronic condition during childhood and adolescence.

**Objectives:**

1) Estimate the risk of non-infectious chronic conditions associated to syphilis, zika, dengue and chikungunya during pregnancy and explore if the risk varies by timing during pregnancy when the infection is acquired (first, second or third trimester) and severity (such as severe or mild dengue); 2) Investigate whether in uterus exposure to maternal infection affects the growth pattern of children and adolescents; 3) Examine the extent to which the relationship between maternal infection and non-infectious chronic outcomes are mediated by intrauterine growth restriction and preterm birth.

**Methods:**

We will compare health outcomes and growth trajectories of children and adolescents born to mothers with and without specific infections during pregnancy using conventional multivariable regression in the whole study population, in a within sibship design, using the subgroup of offspring with at least one sibling who is not exposed to the infection, and negative control outcome. Then we will decompose the direct and mediated effects (by preterm birth and small for gestational age) of maternal infection on chronic disorders.

**Results and Conclusions:**

The results from this study will advance our understanding of the relationship between infections during pregnancy and chronic disorders, with widespread implications enabling targeting of critical points along the path from in utero exposure to outcomes to avoid or mitigate illness and disability over the life course.

## Plain language summary

It is not clear whether when moms get an infection during pregnancy, it might affect their child's long-term health. This means that we do not know whether we could prevent some chronic conditions by screening for and treating infections during pregnancy or by monitoring and treating the child after their birth. In this study, we are going to look at whether infections affecting low—and middle-income countries, like syphilis, neglected tropical diseases such as dengue, zika, and chikungunya, might make children more likely to develop cancer, neurological problems, autoimmune diseases, or obesity when they grow up. We will also check if these risks are connected to things like being born too soon or small. We will use a unique data set from Brazil that follows people from birth (the CIDACS birth cohort). We'll compare the health and growth of children from infancy to teenage years whose mothers had certain infections during pregnancy with those whose mothers did not. The findings from this study will help us learn more about how infections during pregnancy impact the health of children. This knowledge could help us find ways to prevent or lessen the effect of illness and disability throughout a person's life, starting from before they are even born.

## Introduction

The prenatal period is highly sensitive for early development of the brain and of other organs ^1^ and maternal infection during pregnancy can causes disturbances that might contribute to the causal chain of events leading to subsequent development of chronic illness ^2^. Discussed mechanisms include the direct effects of the infectious organism on the developing fetus and exposure to maternal inflammation due to the secretion and circulation of immune-mediated components, such as cytokines, crossing the placental barrier and enter the fetal environment ^3^. These processes may involve alterations in gene expression via epigenetic modifications ^2, 4^. Additionally, such infections might cause a transient depletion of nutrients, e.g. iron, limiting the availability of essential elements necessary for normal fetal development and growth ^2^. Moreover, these mechanisms might result in intrauterine growth restriction and preterm birth, which have been associated with non-infectious chronic condition later in life ^5, 6^

Empirical data on the long-term repercussions of maternal infections during pregnancy on offspring health has accumulated over the last decades but uncertainty remains. Most of the evidence stems from studies conducted in high income countries focusing their research on specific types of infection such as cytomegalovirus, influenza and herpes virus or general systematic infections (e.g. urinary tract infection) ^7^. Another constraint of some studies is the small sample sizes ^8– 10^, and/or limitations in accurately assessing infection during pregnancy ^7^. They commonly rely on maternal self-reported disease or hospital records ^11– 13^. While self-reporting can capture a broader range of infections, it is susceptible to recall bias. Conversely, hospital records are biased towards severe diseases. Furthermore, associations between maternal infections during pregnancy and subsequent offspring health could be fully or partially explained by unknown or unmeasured family level confounding factors, such as socioeconomic position and genetic variation. In addition to these methodological limitations, the multifaceted nature of maternal infections underscores the necessity to independently explore the impact of various factors, including the type and timing of infection occurrence during pregnancy, the severity of the infection, and treatment availability (e.g., administration of antibiotics for syphilis treatment), given their potential differential effects on the risk of adverse childhood and adolescent outcomes.

In this study, we will investigate the relationship between infections and offspring health, focusing on four infections that impose a high burden in low- and middle-income countries, which have been overlooked by the current literature. Specifically, we will examine infections known to cause severe congenital complications, such as syphilis and Zika, as well as viral infections with documented neurotropism and/or linked to adverse birth outcomes. The latter category includes dengue and chikungunya, for which we have available data. In the case of syphilis during pregnancy, the current literature primarily focuses on congenital syphilis, and the long-term consequences have not been appropriately studied ^14^. Arthropod-borne viral diseases have attracted interest in recent years due to their increasing incidence and expanding geographical area as a direct impact of climate change ^15^. The recent zika epidemic highlighted the potential teratogenic effects of this virus, including severe central nervous system anomalies that constitute Congenital Zika Syndrome. Any long-term consequences in offspring prenatally exposed without symptoms at birth are just now being described, with some ^16, 17^ but not all studies reporting developmental delays in children ^18^. Studies of the effects of prenatal exposure to chikungunya infection have suggested a neurodevelopmental risk for children infected during the intrapartum period ^19^. Research on adverse outcomes of prenatal dengue exposure has focused on birth outcomes and demonstrated an effect on stillbirth ^20^ and spontaneous abortion ^21^.

The selected outcomes for this study are a combination of conditions with either high morbidity or mortality during childhood and adolescence, for which a potential relationship with prenatal exposure to infection has been suggested. Infection during pregnancy has repeatedly, although not consistently, increased the risk of neurological ^22– 25^ and autoimmune disorders ^26^. However, further information on candidate pathogens, timing, and clinical features of infections is lacking. An increasing body of evidence for specific cancer onset during childhood and adolescence (e.g., leukemia, lymphoma) has demonstrated that abnormal immune responses to common infections may trigger these diseases ^27^. Although less is known concerning in utero exposure to maternal infection during pregnancy, which could lead to chromosomal or immunological changes and potentially cause cancer ^28^. In addition, maternal infection can cause a congenital malformation that has been associated with childhood cancer ^6, 29^. Regarding growth patterns, most of the literature has focused on maternal antibiotic use during pregnancy or labor, which a few studies have reported to be associated with an increased risk of obesity in childhood ^29, 30^. The most prominent candidate explanation for a causal link between maternal infections during pregnancy and childhood/teenage obesity proposes a causal pathway via changes in gut microbiota detrimental to the maintenance of normal metabolic processes and homeostasis ^31^. One study attempted to disentangle the effect of antibiotic use from underlying bacterial infections and found a small but significant increased risk ^32^ due to infection.

The Study of Early-life Exposures During Developmental Stages (SEEDS) is designed to explore the relationships between prenatal and early life exposures on health outcomes throughout the life course. While SEEDS has a broader research scope, this program protocol will specifically focus on the effect of prenatal exposure to infectious diseases on offspring. Adopting the 'life course' conceptual model, SEEDS recognizes that risk factors experienced during pregnancy and childhood can significantly influence developmental trajectories, potentially elevating the risk of various diseases.

## Aims and objectives

This proposal aims to investigate whether syphilis, zika, chikungunya or dengue during pregnancy can increase offspring's risk of developing neurologic disorders, auto-immune conditions, specific cancers or abnormal growth patterns during childhood and adolescence.

1. Investigate whether syphilis, zika, chikungunya or dengue during pregnancy are associated to neurologic disorders, auto-immune conditions and specific cancers and explore if the risk varies by timing during pregnancy when the infection is acquired (first, second or third trimester) and severity (such as severe or mild dengue);
2. Investigate whether in uterus exposure to syphilis, zika, chikungunya or dengue affects the growth pattern of children and adolescents;
3. Examine if the relationships between syphilis, zika, chikungunya or dengue during pregnancy and neurologic disorders, auto-immune conditions, specific cancers and abnormal growth patterns are mediated by intrauterine growth restriction or preterm birth.

## Proposed methods

### Study population and period

For this study, we will use the Center of Data and Knowledge Integration for Health (CIDACS) Birth Cohort, a Brazilian population-based cohort derived from linked data (SINASC-CadUnico), developed to investigate the relationships between prenatal and early life events on health-related outcomes for infants, children, and adolescents ^33^. The CIDACS Birth Cohort includes Brazilian live births whose families applied for any social government benefit (poorest half of the Brazilian population). This includes approximately nineteen million live births between 1 January 2007 and 31 December 2018, with further updates expected during the study period.

### Data sources

This study is going to use routine data from the Brazilian Information System, which has a long tradition of collecting administrative data; its quality is well documented and constantly improving ^34– 36^. Several liked datasets will be used: SINASC (Live Birth Information System), CadUnico (Social records); SINAN (Information System for Notifiable Diseases); SIH (Hospitalization Information System); SIM (Information System of Mortality); SISVAN (Food and Nutrition Surveillance System) ( Table 1).

**Table 1.** Description of datasets that will be used in this proposal.

Data source	Data type	Approximate coverage	Examples of relevant variables	Used for:
SINASC	Live birth records in Brazil	97% of live births	Newborn characteristics (sex, Apgar scores, birth weight, ICD-10 coded congenital malformation), maternal characteristics (name, age, marital status, education, race/ethnicity, place of residence), paternal characteristics (name, age), perinatal information (reproductive history: parity, abortions, stillbirths, gestational duration, delivery mode, number of fetuses, antenatal care attendance), location of birth	The intersection between these two datasets created the CIDACS Birth Cohort
CadUnico	Social records from over 130 million individuals aged ≥16 years whose families applied for social assistance in Brazil	>50% of population	Location of residence (municipality, region, urban or rural), living conditions (housing, water sources, electricity, sewage, waste, household density), socioeconomic characteristics (education, employment, income), demographics (age, sex, race/ ethnicity), social protection program participation and conditionalities	
SINAN	Records of communicable diseases of interest in the country	----	It is disease specific. Onset of symptoms, date of birth, patient name, sex, address, laboratory confirmation, symptoms and treatment	Exposure
SIH-SUS	Hospitalization admission records	75% of hospitalizations in the Brazilian National Health System	Cause of hospitalization (ICD-10 code), duration, costs, and date of hospitalization, type of hospital	Outcomes
SIM	Death records in Brazil	75–95% of Brazilian deaths, with some geographic heterogeneity	Cause of death (ICD-10 code), characteristics of the deceased (dates of birth and death, name, name of parents, sex, race/ethnicity, birth weight for infants), place of death, characteristics of mother of deceased children (maternal name, age, marital status, education, occupation, race/ethnicity, number of births, place of residence, length of gestation, number of previous stillbirths or abortions, type of delivery)	Outcomes
SISVAN	Anthropometric and food consumption records	30% of children <5 years attending primary care in the Brazilian National Health System, with heterogeneity across populations	Date of birth, age, sex, race/ethnicity, anthropometrics, breastfeeding, complementary feeding, consumption of healthy and unhealthy foods	Outcomes

1. SINASC (birth registry): This system is updated using the registration of live births. This is a legal document completed by the health professional who assisted with the delivery (whether at home or facilities).
2. CadUnico (social registry): This is an extensive questionnaire with information on the household and each individual. The CadUnico database is electronic and longitudinal and has detailed information on demographic, social, environmental, and economic features on named individuals grouped into families. The information is renewed periodically as long as the person is a candidate to receive one of the several Brazilian government social protection programs.
3. SINAN (infections registry): In Brazil, compulsory notification is required for a list of diseases following Ordinance No 264/2020, including all the infections that will be studied in this proposal. The form is disease specific and suspected cases must be reported to the Epidemiological Surveillance service on a specific numerated notification form available in any local health facility. It can be filled in by any health professional who suspects the disease. The Epidemiological Surveillance Centre then investigates to confirm or discard the suspicion based on the Brazilian definition of clinical-epidemiological cases and/or laboratory results. The type of laboratory test will depend on the disease ^37^. Dengue, for example, could be confirmed by IgM antibodies detection by enzyme-linked immunosorbent assay (ELISA), viral Ribonucleic acid (RNA) detection via Polymerase chain reaction (PCR), Nonstructural protein 1 (NS1) viral antigen detection, or positive viral culture; type and result of tests conducted are included in the record. The case must be confirmed or discarded within 60 days. Suspected cases are discarded if one of three conditions are met:1) negative laboratory test (once confirmed that the sample was collected in the appropriate time); 2) confirmation of differential diagnoses; 3) case without laboratory confirmation and with signs and symptoms compatible with another pathology. After confirmation, information on the clinical progress of the case is collected and then, it is classified into clinical categories if applicable: for example, syphilis can be classified as primary, secondary, latent and tertiary syphilis. The sensitivity of the information system to detect this infectious varies by type and we will investigate the impact of potential misclassification of exposure during the study.
4. SIH (hospitalization registry): All hospitalizations’ admissions financed by the Brazilian National Health System are recorded in this system (about 75% of the Brazilian population uses the universal Brazilian system) with this percentage likely higher among the poor population registered in CadUnico. The cause of hospitalization is reported as the diagnosis that motivated the hospitalization (coded using the ICD-10 code). It is filled by the health worker that requested the admission, normally a physician, with few exceptions such as childbirth, in which a midwife can request admission.
5. SIM (death registry): Death-related information will be obtained from the Mortality Information System, which records all Death Certificates. The death certificate is a legal document that must be completed by the physician responsible for clinical care, an assistant or another practician from the institution who attests to the cause of death (using the ICD-10 code) ^38^.
6. SISVAN (food and nutrition registry): will be used to obtain child and adolescent growth. Data from this system has information on anthropometric measurements, including weight and height, food consumption, breastfeeding and complementary feeding practices. The national population coverage of SISVAN ranges between 10% and 15%, mainly among children and adolescents. For those registered in the cash transfer program Bolsa Familia, who are also enrolled in the CadUnico, the SISVAN coverage varies from 57% to 86% ^39^

### Data linkage

To link the datasets, the CIDACS team have used the CIDACS-RL, a novel record linkage tool developed to link big administrative data based on identifiers, such as name, sex, age or date of birth, mother's name, and the municipality of residence ^40^. The CIDACS-RL applies indexing and searching algorithms implemented in the Apache Lucene solution as the blocking strategy. The indexation strategy allows the CIDACS-RL to search for the most similar records from the indexed dataset for each record in the main dataset and submit them to the pairwise comparisons step. Candidate linking records are ordered by the similarity scores, and only the comparison pair with the highest score is retained as a potential link. All remaining candidate records were discarded. The linkage strategy is detailed described in Barbosa *et al.* (2020) ^40^. The linkage quality has been continuously assessed, and accuracy varies according to linked datasets.

### Exposure definition

Information on exposure to syphilis, zika, dengue, chikungunya will be assessed via record linkage between the cohort and the Information System for Notifiable Diseases (SINAN). We will use clinical and laboratory-confirmed cases and conduct sensitivity analyses, including only those laboratory-confirmed, and if applicable, analyses can also be performed according to disease severity and treatment and timing of infection. The period for which data are available for each disease will vary according to data availability and how long disease has been circulating in Brazil, with data available for syphilis and dengue from 2007 and from 2015 for chikungunya and zika.

### Outcome definition

The outcomes will be assessed by record linkage of three different information systems depending on the disorder (SIH-hospitalization data, SIM-mortality data, and SISVAN-nutrition surveillance).

1) Neurologic disorders (cerebral palsy, epilepsy, autism, motor neurone disease), auto-immune conditions (type I diabetes, membranous nephropathy, inflammatory bowel disease, autoimmune hepatitis and juvenile arthritis), and specific cancers (lymphoma, leukemia and neuroblastoma) will be assessed by linking the cohort with hospitalization and mortality data. They will be identified using the ICD-10 code. We know that not all disease episodes will lead to hospitalization; however, due to population size, we predict enough events.

2) Growth patterns will be identified by linking the cohort with nutrition registry data (nationwide coverage reaching up to 43% among children ^41^). We will use data on weight and height to calculate body mass index (BMI) and plot these measurements on growth charts for boys and girls. To describe trajectories in child weight, height and BMI, we plan to test three different classes of models: latent class growth, broken stick and Super Imposition by Translation And Rotation (SITAR) models ^42– 44^. The latent-class growth models allow longitudinal data to inform clusters of individuals following different patterns of change over time, and they account for varying distances between the measurements. The number of trajectory groups can be selected based on Bayesian information criteria. The broken stick models are particular case of the linear mixed model ^44^. They summarize irregular individual trajectories, that is, non-equidistant measurements over time, by estimates made at a prespecified time interval. The models were initially developed to facilitate the statistical analysis and the testing of critical ages in the onset of childhood obesity, but several other applications are possible. Finally, we plan to test the SITAR models ^42^. These models describe each trajectory in three biologically interpretable parameters: size, time and velocity. By using them, we may estimate each child’s variation from the curve expressed by child-specific random effects estimates for absolute amount, timing and acceleration. Children and adolescents will be classified as wasted (weight-for-height Z-score (WHZ) < -2), stunted (height-for-age Z-score < -2), overweight or obese (WHZ > 2).

### Statistical analyses

Each pair of infections and non-infectious outcomes will be investigated separately. We will then select all potential confounders irrespective of whether or not there is data available. Then, Directed Acyclic Graphs (DAGs) will be constructed, including the a *priori* defined set of confounders and mediators. These DAGs will be used to explore residual confounders and to inform sensitivity analyses ^45, 46^. The consideration of potential confounders will be informed by the existing literature and the knowledge of the investigator and collaborators.

The results from different epidemiological approaches will be triangulated to strengthen causal inference. The idea behind triangulation is that when we compare different approaches with assumed unrelated sources of bias ^47^, in our study, we will be able to compare the results and if they point to the same answer, this strengthens causal inference. The key sources of bias will be explicitly acknowledged for each approach when comparing results. Where results point to different answers, we plan to understand the key sources of bias to help direct the research. The methodological approach implemented will vary according to exposure characteristics. Below, we present the main epidemiological approaches planned to answer objectives one and two.

1. Multivariable analyses in the whole study population: We will use multivariable time-to-event models adjusting for the set of confounders to assess each exposure and outcome, comparing children born to mothers with and without notified infection during pregnancy. Specifically for syphilis and zika we will have two exposed groups, those exposed with confirmed congenital infection (congenital syphilis and congenital zika syndrome) and those exposed without symptoms at birth. When appropriate, this will be repeated exploring variations in risk by timing of the disease during pregnancy and by severity of the disease. This approach, however, may be susceptible to residual confounding.
2. Multivariable analyses within sibling groups: We will include participants with at least one sibling in the cohort and compare results between siblings who are discordant for the exposure (i.e. the sub group of participants where the offspring have at least one sibling and at least one sibling was exposed to the infection in utero and the other was not). We will fit semi or parametric survival models comparing the outcomes of siblings within nuclear families. This approach will adjust only for offspring sex and birth order since other factors will be stable within families. Robust standard errors will be used to account for familial clustering at the population level. These matched analyses can control for observed, unobserved family level confounding, such as socioeconomic position and genetics. It has been very powerful to examine the effects of any in utero exposure on outcomes assessed several years later since correlation within siblings is stronger for the confounders than for the exposure of interest during the pregnancy ^47^. However, this approach has limitations. Bias due to misclassification and individual level (as opposed to family) confounding is expected to be greater in within-siblings studies than when comparing unrelated individuals. Furthermore, they can be biased by selection, as only participants with at least one sibling are included, and contamination, where the exposure or outcome in the first sibling influences the outcome or exposure in subsequent siblings, can occur.
3. Cross-context comparisons: Brazil is a remarkably diverse country, encompassing a wide range of social and environmental factors. Our aim is to investigate whether different Brazilian regions present varying confounding structures for each association under investigation. If this assumption holds true, we will conduct stratified analyses. The primary potential source of bias in this approach is the possibility of differential misclassification of infections during pregnancy and non-infectious chronic disorders across regions.
4. Outcome negative control: This approach will select a different condition as a negative control (e.g. car accident). The outcome selected should be likely influenced by the same confounders but would not plausibly be influenced by uterus exposure to infection. There might be differences in the sources of bias between the real assessed conditions and negative control outcome (car accident), and we will explore this issue by investigating the association of observed confounders with the negative control exposure. In this approach, we would expect an unbiased negative control study to be null and the real study not null if there is a true causal effect. However, the negative control study may show a weaker association (than the real study), suggesting some bias in the real study, and we can use this information as an indicator of the extent of bias in the real study ^47^. This approach is particularly useful to detect unmeasured confounding, which can be a limitation of this proposal.

The results provided by all these different approaches will give a complete picture of the relationship between each selected maternal infection during pregnancy and the onset of specific non-infectious chronic disorders during childhood and adolescence.

In this study, we will conduct multiple comparisons, increasing the probability of false positivity. To adjust for multiple testing, we plan to use the false discovery rate (FDR), which has recently been proposed as an alternative metric to family-wise error rate tests. The FDR has been shown to have greater power to detect true positives while still controlling the proportion of type I errors at a specified level ^48^.

To answer objective three, we will implement causal mediation methods to explore the complex causal pathway between infection during pregnancy and the studied outcomes. There is well-established literature about the association between maternal infections during pregnancy and small for gestational age ( *proxy* of intrauterine growth restriction) and preterm birth, which are the mediators of these analyses. The simplest way to estimate the direct (number of outcomes attributed to infection during pregnancy direct path) and indirect (number of outcomes mediated through small for gestational age and prematurity) effects of maternal infection on non-infectious chronic outcomes, is to estimate relative risks with and without adjustment for potential mediating variables. However, simple adjustment for the mediator can give misleading results if there are confounders of the mediator-outcome relationship. In addition to standard regression methods, we will use statistical approaches, such as inverse probability weighting (IPW) based on the counterfactual framework ^49^, to decompose the direct and indirect effect of maternal infection during pregnancy and non-infectious chronic disorders in children and adolescents.

## Ethics and dissemination

Ethical approval to set up the CIDACS Birth Cohort has been obtained by the Federal University of Bahia's Institute of Public Health Ethics Committee (CAAE registration number: 18022319.4.0000.5030) and the London School of Hygiene & Tropical Medicine reference number 22817. We also obtained specific approval covering the objectives presented here, by the Gonçalo Moniz Research Center - CPqGM/ FIOCRUZ/ BA (CAAE registration number: 71054923.8.0000.0040) and the London School of Hygiene & Tropical Medicine reference number 29560.

All data linkage procedures have been conducted in a physically and virtually secure environment and follow strict internal information security measures to ensure data privacy and confidentiality at CIDACS. After linkage, the databases have been fully de-identified: all identifiable elements used for linkage will be removed, and all precautions will be adopted to prevent re-identification The final linked and de-identified datasets will receive a Digital Object Identifier (DOI), and the complete specification of how the dataset was created will be made available upon request. The analysis will be performed in the CIDACS data analysis environment, a safe virtual infrastructure that provides remote data access (for London-based researchers) and analysis tools via Virtual Private Network (VPN).

By identifying the effect of in uterus exposure to each specific infection on high-morbidity or high-mortality non-infectious chronic disorders that consume health care resources, the outputs of this study can be used to estimate attributable fractions – which will give an idea of the effect of preventing these maternal infections during pregnancy would have on the burden of these non-infectious chronic outcomes. Also, the increasing awareness of the effects of maternal infection on non-infectious chronic outcomes may offer guidance for improving public health interventions such as vaccinations, antibiotics and/or antiviral therapy, and dietary adjustments. These interventions might help to minimize the incidence of non-infectious chronic disorders beyond what has been achieved by public health recommendations for maternal–fetal health that are already in place. We expect there to be considerable attention in these findings in light of the strong interest in the effects of maternal infection during pregnancy on the health of children and adolescents.

The research results will be disseminated through peer-reviewed publications. We anticipate publishing high-impact articles and presenting them at conferences to communicate the key findings and scientific advances. Additionally, we plan to share these findings with policymakers editing policy briefings and the public through engagement with LSHTM and CIDACS media channels, such as contributions to written media, as well as through community engagement offices, including participation in science festivals.

## Data Availability

No data are associated with this article.
