# Supplementary material for: A nationwide longitudinal investigation on the role of prenatal exposure to infectious diseases on the onset of chronic conditions in children and adolescents in Brazil
Source: Wellcome Open Res. 2024 Oct 17;9:320. Originally published 2024 Jun 19. [Version 2] doi: 10.12688/wellcomeopenres.22430.2 (PMC11489840; doi:10.12688/wellcomeopenres.22430.2)
Supplement: Peer review history

# Reviewer report: Reviewer response for version 2

**DOI:** 10.21956/wellcomeopenres.25534.r107346

**Published:** 2024-12-02

**Authors:** Suan-Sin Foo

**Affiliations:** 1 Lerner Research Institute, Cleveland Clinic, Cleveland, USA

**General comments:**

 The study by Paixao, et al., entitled “ **A nationwide longitudinal investigation on the role of prenatal exposure to infectious diseases on the onset of chronic conditions in children and adolescents in Brazil**” aim to investigate whether syphilis, zika, dengue and chikungunya during pregnancy can increase the risk of the offspring developing a noninfectious chronic condition during childhood and adolescence Considering that infection during the pregnancy can impact in numerous ways the pregnancy itself and also the fetus development during the pregnancy and after birth, this is an extremely important topic to be investigated. The authors write a protocol to compare health outcomes and growth trajectories of children and adolescents born to mothers with and without specific infections during pregnancy. This is the second revision of the manuscript where the authors addressed all the comments made in the previous revision and made all the adjustments. Small adjustments may be necessary to improve the clarity of the protocol designed by the authors.

 Please see comments listed below.

**Major comments:**

- Will the authors take in consideration and stratify according to the geographic area, family income, maternal age (need to be stratified), number of pregnancies, and possible other cases of delayed neurodevelopment in other siblings due to infection or genetics condition
- Would be interesting to include in the manuscript an experimental design how would be the work flow including the exclusion and inclusion criteria to facilitate the readers to understand the protocol that is being proposed.

**Minor comments**

- Pregnant woman go thought prenatal screening for viral infections can help prevent the transmission of viruses to the infant. Would not be interesting to include in this protocol the results from the previous exposure to Z-STORCH and other arboviruses?  
- In the introduction section could be used to show the importance of viral infection in the pregnancy specifically to the viruses choose in this study. Make clearer why the authors choose specifically ZIKV, Syphilis, CHIKV and DENV, and not included the others virus from Z-STORCH. The authors already included a small section that explain superficially the problem, but can be improved.

Is the study design appropriate for the research question?

Partly

Is the rationale for, and objectives of, the study clearly described?

Yes

Are sufficient details of the methods provided to allow replication by others?

Partly

Are the datasets clearly presented in a useable and accessible format?

Yes

Reviewer Expertise:

Pregnancy research, infectious diseases, virology and immunology.

I confirm that I have read this submission and believe that I have an appropriate level of expertise to confirm that it is of an acceptable scientific standard.

# Reviewer report: Reviewer response for version 2

**DOI:** 10.21956/wellcomeopenres.25534.r112835

**Published:** 2024-11-25

**Authors:** Marisa M Mussi-Pinhata

**Affiliations:** 1 Department of Pediatrics, University of São Paulo, São Paulo, Brazil

The manuscript contains the description of a well-written research project in which the main objective is to search for associations between fetal exposure to prevalent maternal infections (syphilis,  zika, dengue, or chikungunya) in a tropical country (Brazil) and

 the offspring develop a non-infectious chronic condition during childhood and adolescence. Other research questions will also be explored. They clearly describe the rationale for the study. The study intends to approach the problem in a population-based way. They will use several population data sets available in Brazil and a data integration center to do so. They give details of the methods and complex analysis. This innovative approach has the advantage of encompassing a high number of infants exposed to maternal infection, and it is promising to bring new knowledge to the field.  However, potential bias may be included, such as: 1) many maternal infections that are asymptomatic and undiagnosed will be missed; 2) The CadUnico (social records) only contains data from families living with less than half minimal wage registered in the social programs of the Brazilian federation. The authors should mention this limitation of their data approach.

Is the study design appropriate for the research question?

Yes

Is the rationale for, and objectives of, the study clearly described?

Yes

Are sufficient details of the methods provided to allow replication by others?

Yes

Are the datasets clearly presented in a useable and accessible format?

Yes

Reviewer Expertise:

Pediatric Infectious Diseases.

I confirm that I have read this submission and believe that I have an appropriate level of expertise to confirm that it is of an acceptable scientific standard, however I have significant reservations, as outlined above.

# Reviewer report: Reviewer response for version 2

**DOI:** 10.21956/wellcomeopenres.25534.r105818

**Published:** 2024-10-18

**Authors:** Anna Suleri

**Affiliations:** 1 Erasmus University Medical Center, Rotterdam, The Netherlands

I thank the authors for their response and revisions. I think it has improved the analysis plan and look forward to seeing the results.

Is the study design appropriate for the research question?

Yes

Is the rationale for, and objectives of, the study clearly described?

Yes

Are sufficient details of the methods provided to allow replication by others?

Yes

Are the datasets clearly presented in a useable and accessible format?

Not applicable

Reviewer Expertise:

NA

I confirm that I have read this submission and believe that I have an appropriate level of expertise to confirm that it is of an acceptable scientific standard.

# Reviewer report: Reviewer response for version 1

**DOI:** 10.21956/wellcomeopenres.24711.r87458

**Published:** 2024-08-12

**Authors:** Anna Suleri

**Affiliations:** 1 Erasmus University Medical Center, Rotterdam, The Netherlands

In this study by Paixao et al, the authors aim to explore the role of prenatal exposure to infectious diseases and the onset of chronic conditions in children and adolescents. I believe this is an important topic and that this is a comprehensive study. The protocol is well written, provides many details and I compliment the authors for their pre-registration. I have several comments and questions:

1. Please provide more information on the exposure definition:
  - Please provide details on the clinical definition
  - Please provide details laboratory definition (e.g., which assay)
  - Please provide information what sensitivity analysis will be conducted and what the rationale is
  - Please provide information how disease severity will be coded – as I see that treatment is mentioned separately
  - Please provide information how the effect of timing of infection will be measured (e.g. in individual models, G-computation models or mutually adjusted models? And how often is it measured, e.g. once per trimester?
  - It may be interesting to also conduct sensitivity analysis for those exposed to multiple viruses to examine if there is a dose-response relationship between number of viruses prenatally exposed and offspring outcomes
2. Will the outcomes be measured individually, or will a composite score be made? Is there also a way to get information from the GP or subclinical traits of e.g. autism?
3. Will clusters be made for the outcomes based on growth models and will these be placed within the regression models? If there is repeated measure data for the outcome, it may be worth considering performing mixed-effects models that allows to adjust for intra-individual confounding variables.
4. How will the authors deal with missing data in the exposure, covariates and outcome? Will they for example impute part of the missing data, and if so how?
5. Please provide the statistical packages and program the analyses will be conducted.
6. I compliment the authors for adjusting for multiple testing. Please provide the exact number of tests across the aims for which FDR will be applied.
7. I compliment the authors for aiming to create a DAG. Would it be possible to add this to the analysis plan with the exact confounders that will be used?
8. To adjust for selection bias to some extent, did the authors consider inverse probability weighting?
9. Please provide a power analysis based on the given sample size, what the smallest possible effect is the authors are powered to find.
10. Please provide a reference for the cut-offs for the weight-for-heigh Z-score. Did the authors consider calculating a SDS for BMI? And is this based on the WHO reference curves or another curve?
11. What will be the cut-off for the BIC to select the number of trajectory groups for outcome definition part 2? And what will the authors too if the model fit remains poor? And how will the authors ensure (clinical) interpretability of the number of groups based on the growth curves?
12. For the statistical analysis part 2, the within sibling group analyses, I tend to disagree with the authors that only sex and birth are the factors differential within families. Different siblings may for example have a different gestational age at birth, mode of delivery or birth complications which are important confounders in this specific study.
13. How will the matching take place in statistical analysis step 2? Will this be based on propensity scores for example?
14. Objective 3 – statistical analysis: which statistical package in which program will be used to apply the causal mediation analysis and how will the underlying assumptions be tested? What will be done of these associations are violated? And will the mediator be measured continuously or categorically?
15. For all exposure and outcome variables, please provide information, how and when the data is measured, and how it will be coded (e.g. continuously or categorically, and if the latter in which levels)
16. Will the code be made available on GitHub after the study is finished?
17. The authors provide a wide list of outcomes. Other important outcomes may be: other neurodevelopmental disorders such as ADHD, or birth complications (pre term birth, caesarean delivery) or offspring inflammatory outcomes such as neonatal icterus, childhood infections, allergy, atopic dermatitis or asthma, but also cardiovascular development, through e.g. obesity or cardiovascular congenital disorders such as Marfan. I wonder if the authors will be powered enough to find associations with membranous nephropathy, MND and autoimmune hepatitis given that these diseases are rare. It would be informative to conduct a power analysis. 

Is the study design appropriate for the research question?

Yes

Is the rationale for, and objectives of, the study clearly described?

Yes

Are sufficient details of the methods provided to allow replication by others?

Yes

Are the datasets clearly presented in a useable and accessible format?

Not applicable

Reviewer Expertise:

NA

I confirm that I have read this submission and believe that I have an appropriate level of expertise to confirm that it is of an acceptable scientific standard, however I have significant reservations, as outlined above.

# Response

**Published:** 2024-09-29

**Authors:** Enny Paixao Cruz

**Affiliations:** Department of Infectious Disease Epidemiology & I, London School of Hygiene and Tropical Medicine, London, UK

In this study by Paixao et al, the authors aim to explore the role of prenatal exposure to infectious diseases and the onset of chronic conditions in children and adolescents. I believe this is an important topic and that this is a comprehensive study. The protocol is well written, provides many details and I compliment the authors for their pre-registration. I have several comments and questions:

1. Please provide more information on the exposure definition:
  - Please provide details on the clinical definition
  - Please provide details laboratory definition (e.g., which assay)
  - Please provide information how disease severity will be coded – as I see that treatment is mentioned separately
    - Thank you very much we have created a table including information on clinical definition (including severity), treatment and laboratory information used by the Brazilian Ministry of Health, and the definition used in our study.  

-
  - Please provide information what sensitivity analysis will be conducted and what the rationale is
    - We will conduct multiple sensitivity analyses, tailored to the specific exposure and outcome of interest. For instance, regarding arbovirus exposure, we plan to analyze only laboratory-confirmed cases, while for syphilis, analyses can be stratified by VDRL titers. Additionally, we are considering the inclusion of historical control groups for arboviruses introduced later in the country, such as Zika and Chikungunya. This study protocol outlines the general features; however, the analysis plan for each individual study will be developed separately and published on the CIDACS website, under the "SEEDS" section, including all relevant details specific to each exposure and outcome. We have made this clear in the protocol.

-
  - Please provide information how the effect of timing of infection will be measured (e.g. in individual models, G-computation models or mutually adjusted models? And how often is it measured, e.g. once per trimester?
    - The data are derived from administrative databases, and in this context, the time of infection will be determined by the recorded date of symptom onset for arboviruses. The timing of infection will be treated as a time-varying variable. The selection of the model will depend on the estimation of interest. Potential models include, but are not limited to, the Cox proportional hazards model with time-varying covariates, sequential trials, marginal structural models, and G-computation. Landmark models may also be considered where appropriate.
    - For syphilis as there is universal screening and the date of symptoms onset may not be available (it might not have symptoms at all), we will take different approach and timing of infection will not be considered.

1.
  - It may be interesting to also conduct sensitivity analysis for those exposed to multiple viruses to examine if there is a dose-response relationship between number of viruses prenatally exposed and offspring outcomes.
    - This is a nice idea, but it is unlikely that a woman would acquire multiple arboviruses during a single gestational period. However, we are considering including both prenatal and early-life exposures to assess whether a synergistic effect may increase the risk of these conditions in the offspring.
2. Will the outcomes be measured individually, or will a composite score be made? Is there also a way to get information from the GP or subclinical traits of e.g. autism?
  - The outcome will be measured using ICD-10 codes for the cause of hospital admission and deaths. Unfortunately, we do not have GP information, so only more severe cases will be included in the outcome definition.
3. Will clusters be made for the outcomes based on growth models and will these be placed within the regression models? If there is repeated measure data for the outcome, it may be worth considering performing mixed-effects models that allows to adjust for intra-individual confounding variables.
  - No clusters will be formed. The outcomes evaluated will be repeated measurements of height and weight values standardized based on WHO references. Standardized measurements will be classified into nutritional indicators, such as severe underweight, underweight, adequate, or overweight, among others. The Broken-stick model is recommended for assessing irregular individual trajectories and standardized z-score data, providing easily interpretable estimates of childhood growth trajectories (Anderson, Hafen et al. 2019). However, the Broken-stick model does not allow for the inclusion of covariates, meaning factors that may influence growth trajectories were not considered. Given these limitations, we welcome the reviewer’s suggestion to model growth trajectories using mixed-effects models, which allow for adjustment for confounding variables. We have now added to the protocol.
4. How will the authors deal with missing data in the exposure, covariates and outcome? Will they for example impute part of the missing data, and if so how?
  - Exposure and outcome variables are assessed via record linkage. As a result, the absence of linkage is interpreted as the absence of exposure or outcome, meaning there will be no missing data for these variables. For covariates, the mechanisms behind missing data will be carefully considered in each analysis. If the data is believed to be missing completely at random (MCAR), or if the missingness in the exposure or covariates is not driven by the outcome, complete-case analysis may be appropriate. In other scenarios, depending on the analysis, methods such as multiple imputation using chained equations (MICE), pattern mixture models, or growth trajectory models will be considered. The choice of methods for handling missing data, along with any additional techniques such as bootstrapping for calculating 95% confidence intervals, will need to be weighed against the computational demands of working with large datasets. Please provide the statistical packages and program the analyses will be conducted.
  - Potential packages that we are planning to use include, but are not limited to, Mice, tableone, survival, data.table, coxme, lme4, tidyverse, ggplot, dagitty and MatchIt.
5. I compliment the authors for adjusting for multiple testing. Please provide the exact number of tests across the aims for which FDR will be applied.
  - The exact number of tests that will be conducted is not yet known. For each exposure we will compare the different subgroups considered of clinical relevance. For example, for the effect of syphilis exposure on epilepsy, we will compare congenital infection versus those not exposed, maternal (but no congenital) infection versus those not exposed and any infection exposure versus those not exposed. A total of three exposure comparisons. For the example of the impact of syphilis exposure on epilepsy. The analysis confidence intervals will be adjusted to and FDR will account for the three exposure hypotheses we are evaluating.  
6. I compliment the authors for aiming to create a DAG. Would it be possible to add this to the analysis plan with the exact confounders that will be used?
  - Yes, we will include an individual DAG for each plan of analyses with the exact confounders for that relationship, the ones that we are able to control for and even the unmeasured ones. These materials will be produced later during the study's development. In this study protocol, we are giving the readers an overview of the general study. Each individual plan of analyses including DAGs will be publicized in due course in the CIDACS website, under the "SEEDS" section.
7. To adjust for selection bias to some extent, did the authors consider inverse probability weighting?
  - Yes, we will consider the IPW as a method to adjust for confounding and also selection bias. We will include this in the study protocol.  
8. Please provide a power analysis based on the given sample size, what the smallest possible effect is the authors are powered to find.
  -  We deliberately chose not to include a sample size calculation because we agree with the argument presented by Hernan (2022) that the importance of the research question justifies proceeding with imprecise estimate, but rather encouraging the conduct of many observational analyses. Since this study involves an observational analysis of preexisting data, concerns about underpowering should not preclude us from conducting the analysis. This approach allows us to study rare outcomes using valuable, already available data. If the study is ultimately underpowered, we will acknowledge this limitation in the discussion section of the paper. 
9. Please provide a reference for the cut-offs for the weight-for-heigh Z-score. Did the authors consider calculating a SDS for BMI? And is this based on the WHO reference curves or another curve?

- Standard deviations can be computed, but standardized values will be based on WHO reference. Children and adolescents will be classified as wasted (weight-for-height Z-score (WHZ) < -2), stunted (height-for-age Z-score < -2), overweight or obese (WHZ > 2) (de Onis, Onyango et al. 2007).  BMI (kg/m2) will also be calculated. We derived age- and sex-standardized z-scores for BMI using WHO growth reference for under 5 years and 5–10 years.  For children under 5 years, overweight and obesity was defined as weight for height z-scores above two and three standard deviations respectively. The respective cut-offs for children aged 5–10 years were one and two standard deviations.

 

1. What will be the cut-off for the BIC to select the number of trajectory groups for outcome definition part 2? And what will the authors too if the model fit remains poor? And how will the authors ensure (clinical) interpretability of the number of groups based on the growth curves?
  - The BIC (as well as the AIC) are model comparison measures. They do not indicate whether a model is well-fitted; they simply identify the best model among those evaluated. The general approach to assessing model fit is through residual analysis. However, certain characteristics of the evaluated data (large dataset, varied number of measures, and highly irregular measurement times) complicate this analysis. So we are planning to analyse the residual in some random samples of the data and as an alternative approach to assess the quality of the fit using the training and testing method.
  - If the model is poorly fitted, adjustments can be made by modifying the variance component, incorporating a spatial component, or exploring alternative models. The mixed effects model does not stratify the population into distinct groups; instead, it estimates the trajectory of growth and weight gain for specific groups, such as mothers with and without particular infections during pregnancy.
2. For the statistical analysis part 2, the within sibling group analyses, I tend to disagree with the authors that only sex and birth are the factors differential within families. Different siblings may for example have a different gestational age at birth, mode of delivery or birth complications which are important confounders in this specific study.
  - You are completely right, we have added this to the manuscript now.
3. How will the matching take place in statistical analysis step 2? Will this be based on propensity scores for example?
  - Given the large size of our dataset, we sometimes use exact matching. However, when a high percentage of individuals remain unmatched, we resort to propensity score matching as an alternative.
4. Objective 3 – statistical analysis: which statistical package in which program will be used to apply the causal mediation analysis and how will the underlying assumptions be tested? What will be done of these associations are violated? And will the mediator be measured continuously or categorically?
  - Analyses will be conducted in R, utilizing packages such as "mediation" and "medflex." However, these packages do not fully support all types of categorical variables for the exposure, mediator, and outcome. In such cases, custom analysis can still be implemented in R using base functions to manually code the necessary procedures.
  - Mediation analysis relies on strong underlying assumptions about the available information and the relationships between variables. While these assumptions cannot be directly tested, sensitivity analyses can be performed to assess the potential impact of assumption violations.
  - The theoretical model, which serves as the basis for mediation analysis, will be developed in consultation with subject matter experts. Additionally, sensitivity analyses will be used to evaluate the impact of breaking assumptions.
  - A mediator variable, such as gestational age at birth, can be measured as a continuous variable and alternatively categorized to represent preterm birth (yes/no). We will analyze the variable in both forms to assess how these different approaches impact our results.
5. For all exposure and outcome variables, please provide information, how and when the data is measured, and how it will be coded (e.g. continuously or categorically, and if the latter in which levels).
  - The exposures and outcomes will be categorical variables assessed through record linkage. Live births from the CIDACS Birth Cohort, linked with SINAN data from conception to birth, will be classified as exposed to infectious diseases during pregnancy. Dengue exposure can be further categorized into mild dengue, dengue with warning signs, and severe dengue, with all three arboviruses classified based on laboratory testing. Live births exposed to syphilis and Zika can also be further classified as cases of congenital infection. For more details, refer to Table 2, newly added to this protocol. Live births linked to death or hospital admission records containing a relevant ICD-10 code for the specified outcome will be classified as "yes" for the outcome of interest.
6. Will the code be made available on GitHub after the study is finished?
  - Yes, it will be published on the CIDACS website, under the "SEEDS" section.
7. The authors provide a wide list of outcomes. Other important outcomes may be: other neurodevelopmental disorders such as ADHD, or birth complications (pre term birth, caesarean delivery) or offspring inflammatory outcomes such as neonatal icterus, childhood infections, allergy, atopic dermatitis or asthma, but also cardiovascular development, through e.g. obesity or cardiovascular congenital disorders such as Marfan. I wonder if the authors will be powered enough to find associations with membranous nephropathy, MND and autoimmune hepatitis given that these diseases are rare. It would be informative to conduct a power analysis. 

- We fully agree and plan to expand the list of outcomes and exposures investigated by the SEEDS project. This was discussed with our collaborators, and the current list is only preliminary. Other disorders, such as cardiovascular conditions, are of significant interest and will be included in future analyses.

# Reviewer report: Reviewer response for version 1

**DOI:** 10.21956/wellcomeopenres.24711.r87456

**Published:** 2024-08-05

**Authors:** Aja Louise Murray

**Affiliations:** 1 The University of Edinburgh, Edinburgh, Scotland, UK

The paper addresses an important topic with significant public health implications. Whilst there were some minor language errors/typos and  it could benefit from further proof reading, it was also very well-written. The approach of triangulating across different methodologies was a strength. Some suggestions for improvement:

- The arguments for focusing on the specific exposures are well-made and convincing. Can the authors cite any prevalence information for these exposures to further give a sense of the scale of the issue (and further support that the numbers will be sufficient to support their proposed statistical analyses)
- In the exposure definition it is mentioned that timing of infection will be examined in a sensitivity analysis; however, earlier the effect of timing is mentioned as a research question, therefore, should there not be a primary analysis related to this rather than just a sensitivity analysis? Unless timing refers to something else, e.g., month of year. Either way this could benefit from clarification. Otherwise, the research questions mapped well to the proposed analyses.
- Whilst another publication is referenced, it would be helpful to have more details on how the linkage accuracy is assessed and what the levels of accuracies are (even a range or indicative examples would be helpful additions)
- For the mediation analyses, have the authors considered using causal mediation approaches e.g.: Imai, Kosuke et al. (2010 ^1^)

Is the study design appropriate for the research question?

Yes

Is the rationale for, and objectives of, the study clearly described?

Yes

Are sufficient details of the methods provided to allow replication by others?

Yes

Are the datasets clearly presented in a useable and accessible format?

Not applicable

Reviewer Expertise:

Child development

I confirm that I have read this submission and believe that I have an appropriate level of expertise to confirm that it is of an acceptable scientific standard.

## References

[1] : A general approach to causal mediation analysis. *Psychol Methods* .2010;15(4) : 10.1037/a0020761 309-34 10.1037/a0020761 20954780
